# Longitudinal patterns and predictors of suicidal ideation in African American adolescents

**DOI:** 10.1002/ajcp.12663

**Published:** 2023-04-12

**Authors:** Christopher R. Whipple, W. LaVome Robinson, Caleb E. Flack, Leonard A. Jason, Kate Keenan

**Affiliations:** 1 Department of Social Sciences and Psychology, School of Behavioral Sciences and Education, Penn State Harrisburg, Pennsylvania, Middletown, USA; 2 Department of Psychology, DePaul University, Chicago, Illinois, USA; 3 Department of Educational Psychology, University of Wisconsin-Madison, Madison, Wisconsin, USA; 4 Department of Psychiatry and Behavioral Neuroscience, University of Chicago, Chicago, Illinois, USA

**Keywords:** adolescent, African American, latent transition analysis, suicidal ideation

## Abstract

Suicide rates among African American adolescents have increased dramatically. Suicidal ideation is associated with both suicide attempts and completions, thus understanding ideation patterns and predictors in African American adolescents is critical to informing prevention efforts. This study recruited 160 African American ninth grade students. Participants were those students randomized to the control condition of a randomized controlled preventive intervention. Of the 160 participants, 99 completed all assessment points and were included in latent transition analyses. We assessed participants four times: baseline then again at 6-, 12-, and 18-month postbaseline. Constructs of interest for this study included suicidal ideation, depression, hopelessness, and community violence exposure. A 2-class model (i.e., low ideation [LI] and high ideation [HI]) characterized ideation at each time point. A total of 86%–90% of participants were in the LI class in any given time point and 27.3% of participants were in the HI class at least once. Participants in the LI class tended to stay in that class, whereas those in the HI class often transitioned to the LI group. Depression and hopelessness, but not exposure to community violence, predicted HI class membership. Findings suggest that (a) most African American adolescents may experience suicide ideation at some point in time, (b) a concerning proportion of African American adolescents may experience high ideation, (c) high ideation is often time-limited, and (d) depression and hopelessness predict high ideation.

Suicide is an escalating public health threat for African American adolescents. According to recent estimates, suicide is the third leading cause of death for African Americans, ages 15–19 (Centers for Disease Control and Prevention [CDC], 2020). Historically, suicide rates have been higher among European American than African American youth, but this gap has narrowed due to increasing rates among African American youth and declining rates among White youth (Bridge et al., 2015; Price & Khubchandani, 2019). Between 2001 and 2017, nationally representative data revealed that the rate of deaths by suicide grew by 60% for African American adolescent males and 182% for African American adolescent females (Price & Khubchandani, 2019). The rate of reported suicide attempts also has increased among African American adolescents, while decreasing among adolescents from other racial and ethnic backgrounds (Lindsey et al., 2019).

African American adolescents in ninth-grade are at particularly high risk for suicidal behavior. Alarmingly, 12.9% of African American ninth-grade youth report at least one suicide attempt in the past year, compared to 7.7% of their same-grade White peers (CDC, 2019). The rate of self-reported suicide attempt among ninth-grade African American youth also exceeds that of African American youth in later high school years (CDC, 2019). These rates may be underestimates, as cultural norms and stigma within the African American community may perpetuate the misclassification of suicidal behaviors (Ali et al., 2022; Goldston et al., 2008).

Suicidality encompasses suicidal thoughts (e.g., thoughts about killing oneself), behaviors (e.g., attempts), and completions (O’Carroll et al., 1996) and is generally thought to progress sequentially, with ideation preceding attempts (Posner et al., 2011) and deaths by suicide (Large et al., 2021) in adolescence. Seriousness, duration, and frequency of suicidal ideation predicts the transition to a suicide attempt in adolescence (Miranda et al., 2014). Nock et al. (2013) found that most adolescent transitions (i.e., from suicidal ideation to suicide plan and from suicide plan to suicide attempt) transpire within the first year of onset of ideation. Thus, there is an unequivocal need for preventive intervention (a) before the onset of suicidal ideation (e.g., universal preventive interventions) and/or (b) at the earliest onset of suicidal ideation (e.g., indicated preventive interventions).

Efficacious preventive intervention for African American adolescent suicidal ideation requires an understanding of the distinct patterns and predictors of ideation in this population (Robinson et al., 2022). Due to systemic and structural racism, African American adolescents contend with an array of unique contextual risk-factors for suicidal ideation over time (e.g., racial discrimination, community violence; Farrell et al., 2019; Madubata et al., 2022). Consequently, longitudinal patterns and predictors of ideation in these adolescents may differ from adolescents from other racial/ethnic backgrounds (Kim et al., 2019; Robinson et al., 2022). Further, because most African American adolescents will experience discrimination (Smith-Bynum et al., 2014), and those living in urban, low-resourced areas also frequently experience distinct contextual risk-factors for ideation such as exposure to community violence (Copeland-Linder et al., 2011; Richards et al., 2015), a relatively large proportion of African American youth may report suicidal ideation at some point during adolescence. Additionally, compared to adolescents from other racial/ethnic backgrounds, African American adolescents may be more likely to transition from a state of low ideation to high ideation, because they are at greater risk for encountering significant socio-ecological stressors that can lead to elevations in ideation (Farrell et al., 2019; Madubata et al., 2022).

The extant research suggests that African American adolescents have distinct longitudinal patterns of suicidal ideation, relative to adolescents from other racial/ethnic backgrounds, and that identified longitudinal patterns may differ across studies of African American adolescents. Using growth mixture modeling (GMM), Kim et al. (2019) found variability in suicidal ideation patterns from grades 6 through 12, characterized by severity and persistence across racial groups. The three classes that characterized African American adolescents were: no ideation (86%), moderate ideation (10%), and high ideation (4%); the classes for European American adolescents were: no ideation (90%), ideation (6%), and high-decreasing ideation (4%); and for Asian American adolescents, the classes were defined as no ideation (79.5%), increasing ideation (13%), and high-fluctuating ideation (7.5%). For African American adolescents, high levels of ideation were coupled with persistence, a pattern not observed among European American and Asian American adolescents. Specifically, African American individuals in the high ideation group experienced greater severity and longer duration of ideation, as compared to adolescents of other ethnic groups (Kim et al., 2019). The three ideation classes identified by Kim et al. (2019) differed slightly from those found previously by Musci et al. (2016), using longitudinal latent class analysis (LLCA). In an earlier study of urban African American adolescents, ages 11–19, Musci et al. (2016) found two classes of suicidal ideation, specifically those without (92%) and those with ideation (8%). A range of factors may explain differing findings on ideation classes in the studies by Musci et al. (2016) and Kim et al. (2019), including differences in sample composition (e.g., age, gender, socioeconomic status), setting (e.g., urban locale or not), and measurement of suicidal ideation (e.g., single-item vs. multi-item scale). The unique and variable reported patterns of suicidal ideation in African American adolescents warrants further examination and clarification, given the urgency of prevention and the strong link between ideation and both suicide attempts (Musci et al., 2016) and deaths by suicide (Large et al., 2021).

A notable limitation of the above studies (i.e., Kim et al., 2019; Musci et al., 2016) is that their analytic approaches (i.e., GMM, LLCA) allowed African American adolescents to belong to only one ideation trend over time (e.g., no ideation, high ideation). Thus, these studies were only able to identify the overarching trend in suicidal ideation over time; they were not able to identify changes or transitions in individuals’ suicidal ideation classes that occurred from measurement point to measurement point. Prior research in a non-predominantly African American sample suggests that a substantial proportion of adolescents transition in ideation class membership over time (Thompson et al., 2009). Such a transition (or the lack thereof) may be an important marker of suicide risk (Thompson et al., 2009).

Consequently, there is an important need for research examining transitions in African American adolescents’ ideation class membership over time. Such research may provide a granular view of changes in suicidal ideation that allows for more targeted intervention with African American adolescents. Longitudinal research on ideation class membership may identify if there are specific factors that influence subgroups of African American adolescents to transition from a high ideation class to a low ideation class, or from a low ideation class to a class characterized by higher ideation. Identification of factors associated with transitions from high to low ideation may assist in the development of strengths-based preventive interventions. African American adolescents who experience transitions from low to high ideation may benefit from targeted intervention that focuses on building skills for adaptively coping with specific risk-factors for high suicidal ideation (Robinson et al., 2016; Robinson et al., 2021). For example, the often chronic, uncontrollable, and highly stressful nature of community violence exposure for some African American adolescents (Richards et al., 2015) suggests that this exposure may lead directly to suicidal ideation (Farrell et al., 2019) rather than indirectly influencing suicidal ideation through depression (Bennett & Joe, 2015).

Research indicates that conventional risk factors of suicidal ideation may have limited predictive validity for African American adolescents (Robinson et al., 2022). Because of systemic and structural racism, African American adolescents are disproportionately likely to confront distinct socio-ecological stressors that predict suicidal ideation (Assari et al., 2017; Madubata et al., 2022). Additionally, the magnitude and chronicity of contextual stressors for urban, low-resourced African American adolescents (Sanchez et al., 2013), also limit the predictive salience of conventional predictors of suicidal ideation for these youth. For example, the extant research generally supports depressive symptoms as strongly linked to adolescents’ suicidal ideation, but these conclusions derive from samples largely comprised of European American adolescents (Czyz & King, 2015; Prinstein et al., 2008). A less consistent pattern of predictors and correlates of suicidal ideation is observed in studies of African American adolescents. In some studies, strong and positive associations between depressive symptoms and suicidal ideation (e.g., Bennet & Joe, 2015; O’Donnell et al., 2004) were reported, whereas results from other studies showed that suicidal ideation occurred at relatively low levels of depression severity in African American adolescents (Robinson et al., 2016) and in the absence of psychiatric diagnoses, including depression (Joe et al., 2009).

The link between hopelessness and suicidal ideation, on the other hand, is comparatively consistent between African American adolescents and adolescents from other racial/ethnic backgrounds (Molock et al., 2006; Perkins & Hartless, 2002). It is important to note, however, that Lamis and Lester (2012) observed a stronger association between hopelessness and suicidal ideation among African American female young adults than among their European American peers.

Both theory and research advance community violence exposure as a key contextual risk-factor for suicidal ideation, particularly so for African American adolescents (Farrell et al., 2019; O’Leary et al., 2006). Yet, so far, the research is indeterminate as to whether community violence exposure is directly associated with ideation (Farrell et al., 2019) or indirectly associated through other factors such as depression (Bennett & Joe, 2015; Lambert et al., 2008). The Interpersonal Theory of Suicide ([IPT]; Joiner, 2005) posits that an unmet need for social connection (i.e., thwarted belongingness) is a primary driver of suicidal ideation. From the perspective of IPT, community violence may lead to suicidal ideation in adolescents when it creates a threatening environment that disrupts social connections in the community. Left unaddressed, this exposure may lead to a range of mental health concerns, in addition to suicidal ideation, that include anxiety, depressive symptoms, and externalizing behavior (Edlynn et al., 2008; Pittman & Farrell, 2022; Sargent et al., 2020).

In sum, the available studies report a considerable degree of variability in patterns and predictors of suicidal ideation in African American adolescents, thereby accentuating the need for further examination to target prevention efforts. Furthermore, existing research on predictors of suicidal ideation in African American adolescents has been conducted largely using cross-sectional designs. Consequently, for this group of adolescents, the extent to which predictors of suicidal ideation change or remain stable over time is unclear. There is a need for longitudinal research on predictors of ideation in African American adolescents, to inform targets of culturally- and contextually-relevant suicide preventive intervention.

The present study aims to address several important gaps in the research literature. To date, there is limited research examining both longitudinal patterns and predictors of suicidal ideation in African American adolescents. Previous studies that have investigated longitudinal patterns of suicidal ideation in African American adolescents have not examined *transitions* from one suicidal ideation class to another over time, or the likelihood of such transitions occurring, even though such transitions may be meaningful indicators of suicide risk. Hence, the present study uses a latent transition analysis (LTA) of suicidal ideation in African American adolescents to identify: (a) classes of suicidal ideation operative for African American adolescents, (b) change in ideation class membership over time, and (c) predictors of ideation class membership (i.e., depression, hopelessness, and exposure to community violence) at each time point.

## METHOD

### Participants

Participants were ninth-grade students recruited from four public high schools with a predominantly African American enrollment and located in a large Midwestern city. Adolescents included in this study were drawn from the control group of a randomized controlled trial (RCT) of a culturally-tailored, stress-reduction preventive intervention that was intentionally designed for urban African American adolescents. A total of 408 participants were recruited for the RCT, and 160 were randomized to the control condition (i.e., the sample for this study). The participation rate was 73.3% (i.e., recruited students who returned both signed parent/guardian and student assent forms). The average age of study participants was 14.5 years (*SD* = 0.6), and 57.5% were female.

### Procedure

Trained research staff recruited participants in their ninth-grade year at participating public high schools, during school registration activities. Participants completed a brief screening survey to identify elevated suicide risk; those who screened positive received follow-up services at their respective school-based health centers (SBHCs) and their study participation ended. Of the participants who completed screening assessments, approximately 1% of students screened positive for elevated suicide risk. Participants who did not have elevated suicide risk were randomly assigned either to the intervention condition or to the standard care control condition. Participants completed four additional assessments following the screening: (a) baseline (time point 1 [T1]), (b) 6 months after baseline (time point 2 [T2]), (c) 12 months after baseline (time point 3 [T3]), and (d) 18 months after baseline (time point 4 [T4]). Participants received $7 for completing the screening survey and $15 for each of the four assessments, as modest tokens of appreciation. At each time point, participants again completed measures of suicidal ideation. Participants who were identified as at elevated suicide risk were referred to their SBHC for risk assessment and additional support, as necessary. Approximately 62% of control participants who completed the baseline assessment continued in the study to complete all four assessments.

The DePaul University Institutional Review Board and the school district’s Research Review Board approved all study procedures.

### Measures

#### Demographic information

Participants completed a 17-item measure of demographic characteristics that included age, sex, race, household income, parent employment and education, family size, and religious involvement.

#### Suicidal ideation

The Center for Epidemiological Studies—Depression scale (CES-D; Radloff, 1977) appended suicide items (Lewinsohn et al., 1996) were used to measure suicidal ideation. This is a self-report inventory containing 4-items on a Likert response scale from 0 (*rarely or none of the time*) to 3 (*most of the time*) to assess past week suicidal ideation (see Table 1 for items). Responses were summed across items, with higher scores indicating higher levels of ideation. The CES-D appended suicide items have evidenced validity and reliability (Lewinsohn et al., 1996). In the current study, estimates of internal consistency ranged from 0.76–86.

#### Depression

The CES-D (Radloff, 1977) was used to measure depressive symptoms. The CES-D is a 20-item measure, rated on a 4-point Likert scale from 0 (*rarely or none of the time*) to 3 (*most of the time*). Participants reported how often they experienced each symptom within the past week. These depression items did not include items assessing suicidal ideation. Four items were reverse scored before summing items to create a total depression score; higher scores indicated more depressive symptomatology. The CES-D has established validity and reliability (Dierker et al., 2001). In the current study, internal consistency estimates ranged from 0.82–0.86.

#### Hopelessness

The Hopelessness Scale for Children (HSC; Kazdin et al., 1986) was used to measure hopelessness. The HSC is a 17-item self-report inventory. Participants were instructed to respond whether each item represented their thoughts and feelings most of the time. Participants responded to each item as being true or false. The sum of true responses yielded a total hopelessness score, with higher scores indicating greater levels of hopelessness. The HSC has demonstrated validity and moderate test-retest reliability over a 6-week period (*r* = .52; Kazdin et al., 1986). In the current study, internal consistency estimates ranged from 0.74–0.75.

#### Exposure to community violence

The Children’s Report of Exposure to Violence (CREV; Cooley et al., 1995) was used to measure community violence exposure. The CREV is a 25-item, self-report inventory that measures the frequency of past-year exposure, rated on a 5-point, Likert-type scale from 1 (*no, never*) to 5 (*every day*). Items are summed to create a total score, with higher scores indicating greater exposure. The CREV has evidence of construct validity and test-retest reliability (*r* = .75), and internal consistency (α = .78; Cooley et al., 1995). In the current study, internal consistency estimates ranged from 0.93−0.96.

### Analytic strategy

We used LTA to longitudinally examine patterns of suicidal ideation and the impact of individual and contextual factors on suicidal ideation. LTA is a person-centered analysis that allows for the examination of classification changes over time (Lanza et al., 2012). Specifically, in LTA, latent classes of indicators are identified at each time point and the likelihood of transitioning from one latent class to another across adjacent time points is estimated. Additionally, observed covariates can be included to identify how covariates influence classification at each measurement point (Nylund, 2007). We followed a step-by-step model to estimate measurement and structural models (Nylund, 2007). All analyses were conducted using Mplus Version 8.3 (Muthén & Muthén, 1998–2017) and models were estimated using full information maximum likelihood (FIML) estimation. Because the ideation indicators were continuous, we used latent profile analysis (LPA) to estimate measurement models. LPA is a person-centered analysis that identifies latent classifications of individuals based on their responses to multiple continuous indicators (Williams & Kibowski, 2016). In this study, indicators were ordinal responses (i.e., 0 [rarely or none of the time] to 3 [*most of the time*]); however, these were treated as continuous for the purposes of this analysis. Multiple models were evaluated at each time point to determine class structure using several fit indicators, including Bayesian Information Criteria (BIC), Vuong-Lo-Mendell-Rubin Likelihood Ratio Test (VLMB-LRT), and the Bootstrapped Likelihood Ratio Test (BLRT), in addition to the interpretability of class structure. The model with the lowest BIC indicated the best fitting model; VLMB-LRT and BLRT with significant *p* values indicated that the current model was a better fit for the data compared to a model with *k*-1 classes. In a simulation study, Nylund, Asparouhov, et al. (2007) identified the BIC and BLRT as the most consistent indicators of the number of latent classes.

A structural model was estimated using LTA, after evaluating class structure. Transition probabilities were estimated between adjacent time points. Lastly, depression, hopelessness, and exposure to community violence were included in the structural model to determine their effects on classification at each time point.

FIML estimation was used to manage missing data. FIML estimation utilizes all available information to estimate model parameters. As such, cases were retained when they had some data on outcome variables. For the LPA models, cases were excluded only when participants had no data on dependent variables. For the LTA model, cases were excluded when cases were missing data on all dependent variables or when data were missing on covariates.

## RESULTS

**FIGURE 1:**
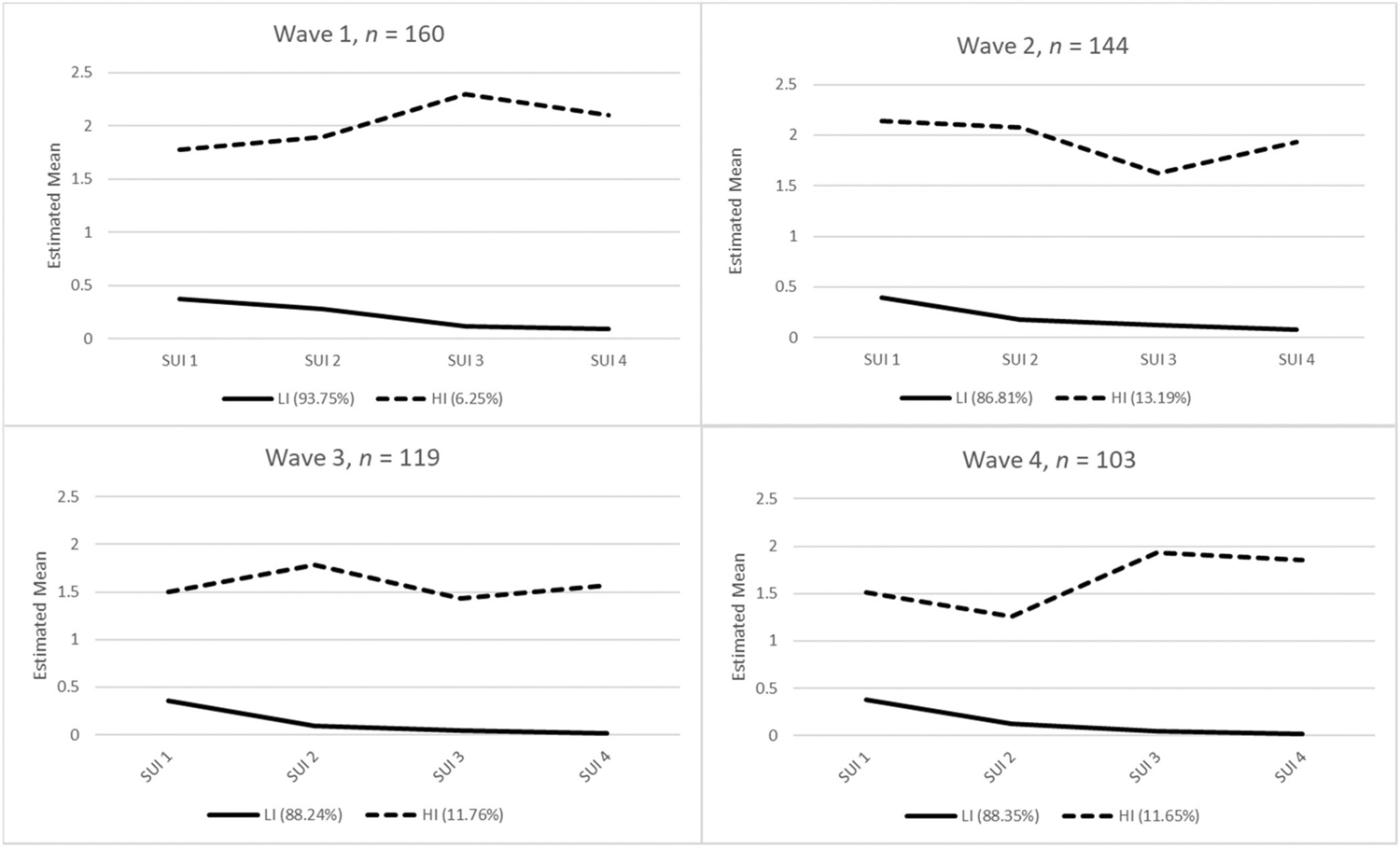
Class structure of suicidal ideation at each measurement wave. SUI1 = “I had thoughts about death;” SUI2 = “I felt my family and friends would be better off if I were dead;” SUI3 = “I thought about killing myself;” SUI4 = “I felt that I would kill myself if I only knew a way;” HI, high ideation group; LI, low ideation group

There were 160 African American participants in the standard care control condition and 99 of these participants had at least some data on suicidal ideation and all data for covariates. The 99 participants were used to estimate LTA models. Samples sizes for LPAs are included in Figure 1. Descriptive statistics for suicidal ideation indicators and covariates are presented in Table 1.

Before conducting LPA and LTA analyses, attrition analyses were conducted. Specifically, participants who completed all assessment points were compared to participants who discontinued participation on demographic characteristics and baseline suicidal ideation. There were no significant differences in age, *t*(158) = 1.54, *p* = .125; gender, *χ*^2^(1, *n* = 160) = 0.09, *p* = .761; or baseline suicidal ideation, *t*(158) = 0.53, *p* = .598.

### Measurement models

We conducted LPAs with the four suicidal ideation items from the CES-D to identify appropriate measurement models for each time point, starting with a 1-class model and up to a 4-class model (see Table 2 for model fit statistics).

At T1, the 2-class model had significant VLMR-LRT and BLRT values and a lower BIC than the 1-class model. For 3- and 4-class models, the BLRT was significant, but the VLMR-LRT was not. Thus, the 2-class model best fit the Wave 1 data. For T2, T3, and T4, the 2-class model had lower BIC than the 1-class model, a significant BLRT, and a nonsignificant VLMR-LRT. In estimating the 3- and 4-class models, the best log likelihood was not replicated. In addition, for T3 and T4, the 3- and 4-class models had at least one latent classification with fewer than 5% of participants. As 3- and 4-class models encountered issues with replication and small *n* within latent classifications, 1- and 2-class models were compared. Due to the lower BIC, relative to the 1-class model and the significant BLRT, the 2-class model was selected as the best-fitting model for T2, T3, and T4, also.

The 2-class models were then interpreted with respect to level of suicidal ideation (see Figure 1). The first class indicated low ideation scores (i.e., “low ideation” [LI]), and the second class indicated higher scores for ideation (i.e., “high ideation” [HI]). At each time point, approximately 90% of participants were classified in the LI class; however, some differences in classification were identified. At T1, only 6.3% of participants were classified in the HI group, but at T2, the proportion of respondents in the HI group increased to 13.2%. At T3 and T4, proportions in the HI group were 11.1% and 12.1%, respectively.

### Latent transition analysis

LTA models were examined to calculate transition probabilities between classes and the influence of covariates on class membership. Before examining the structural model, class structure measurement invariance was tested over time. Full measurement invariance suggests that the number and definition of each class is the same across measurement points. If full measurement variance is assumed, interpretation of classes and transitions are straightforward (Nylund, 2007). As such, full measurement invariance is preferred. To test full measurement invariance, a model fixing indicators to equality across time points was estimated. This invariant model was compared to a freely estimated model with no invariance. The fixed model did not have significantly different fit relative to the freely estimated model, *χ*^2^_diff_ = 26.37, *df* = 24, *p* = .335. Thus, the invariant model was used.

A fully invariant LTA was estimated including covariates. Class membership at each time point and transition probabilities across adjacent time points are included in Tables 3 and 4, respectively. In general, participants in the LI class at an earlier time point were highly likely to be in the LI class at the following time point. However, those in the HI class at T1 were just as likely to stay in the HI class at T2 or transition to the LI class. Similarly, those in the HI class at T3 were just as likely to stay in the HI class at T4 or transition to the LI class. Participants in the HI class at T2 were more likely to transition into the LI class at T3. There were 16 possible transition patterns (see Table 5). Nearly three-quarters of participants were in the LI class at each time point (72.73%). The remaining 27.27% were in the HI class during at least one time point. Last, 11.11% of participants were in the HI class during at least two time points.

We then examined the influence of covariates on class membership by calculating odds ratios with the HI class as the referent group. Specifically, an odds ratio higher than one indicated a greater likelihood of being in the LI group, while an odds ratio lower than one indicated a lesser likelihood of being in the LI group, relative to the HI group. Odds ratios were calculated by Mplus as part of the LTA analysis including covariates. Coefficients for all covariates are provided in Table 6. Only depression and hopelessness were significantly associated with class membership. Higher levels of depression were associated with a lower likelihood of being in the LI group at all time points. At T2, higher levels of hopelessness were associated with a lower likelihood of being in the LI group. Community violence exposure was not significantly associated with suicidal ideation class membership at any time point.

## DISCUSSION

The alarming increase in suicide rates among African American adolescents over the past 20 years (Price & Khubchandani, 2019) amplifies the urgent need to identify both patterns and predictors of suicide risk for this group of youth, to advance effective prevention efforts. The transitory nature of suicidal ideation across adolescence (Thompson et al., 2009) highlights the need to understand how, when, and why adolescents’ suicidal ideation changes. We addressed this need by longitudinally examining suicidal ideation classifications, transitions, and predictors, within a community sample of African American ninth-grade students living in urban, low-resourced neighborhoods.

In the present study, a 2-class model of suicidal ideation, consisting of a low ideation class and a high ideation class, best fit the data at each wave of data collection. An earlier study, Musci et al. (2016), identified two classes of ideation in a sample of African American adolescents (i.e., nonideators vs. ideators), and Kim et al. (2019) subsequently identified a third class of ideators, consisting of moderate ideators. Differences in the number or definition of ideation classes may be attributable to several explanations. First, different analytic techniques may account for some of the discrepancies. Musci et al. (2016) and Kim et al. (2019) utilized longitudinal LLCA and GMM, respectively. These approaches model longitudinal trends in suicidal ideation. In the current study, we identified ideation class structures at each wave and examined transitions in class structures over time. Second, Kim et al. (2019) examined the trajectory of ideation in sixth through 12th grade; however, no measurements were taken between ninth and 12th grade. As such, the gaps in data points in Kim et al. (2019) that would be consistent with the current study’s time points (i.e., measurements in ninth and tenth grade) may reduce our ability to create accurate comparisons in class number or structure. In addition, we note that both the Musci et al. (2016) and Kim et al. (2019) studies utilized data collected at least 10 years before that of the current study. Further, while the findings of the present study are similar to the Musci et al. (2016) study, in that both studies observed two classes of ideation, our tests of transitions across short time periods yielded important information about changes in ideation.

In the present study, participants who initially (Wave 1) evidenced low ideation were unlikely to transition into the high ideation class at any subsequent wave, whereas participants initially in the high ideation class frequently transitioned into the low ideation class at a subsequent wave. Additionally, the low ideation group, on average, reported low scores on ideation indicators, although the average ideation score was not zero as in the Musci et al. (2016) study. The average non-zero ideation score in the low ideation group, which consisted of 87%–90% of participants at any given time point, indicates that some level of suicidal ideation is common among African American youth. In the LI group, nonzero levels of ideation were driven primarily by participant responses to the items, “I had thoughts about death,” and “I felt my friends and family would be better off if I were dead.” These questions highlight passive suicidal ideation, or a desire for death (Van Orden et al., 2010). In the IPT (Van Orden et al., 2010), passive suicidal ideation may manifest when feelings of burdensomeness or thwarted belongingness are present. In the presence of hopelessness, particularly hopelessness about belonging and/or burdensomeness, passive ideation may become active ideation (Van Orden et al., 2010), manifested by intent for suicide (e.g., “I thought about killing myself,” “I felt that I would kill myself if I only knew a way”). In brief, adolescents in the LI group who evidenced low levels of ideation, may be indicating some level of passive ideation. This is a very important and warning finding. Even low levels of suicidal ideation should not be ignored in African American youth, given that stigma (Anderson et al., 2015) and cultural mores (Neeleman et al., 1998) may lead to under-reporting of suicidal ideation intensity, consequently reducing help-seeking (Goldston et al., 2008; Lindsey et al., 2017).

The escalating trend in suicidality among African American adolescents nationally (Lindsey et al., 2019; Price & Khubchandani, 2019) is consistent with our results, showing that the number of African American youth for whom suicidal ideation is absent from their developmental trajectories is diminishing. It is noteworthy that, although the percentage of youth in the high ideation group was approximately 10% in each of the 4 waves, almost 30% of youth were members of the high ideation group at some point during the period of the study. In other words, a third of the adolescents reported ideational patterns associated with high suicide risk, for a 2-week period or more, during their first or second year of high school.

The current findings both support and fail to support previous findings regarding the impact on suicidal ideation of individual and contextual level factors. Prior studies have identified depression (Bennett & Joe, 2015), hopelessness (Molock et al., 2006), and community violence exposure (Farrell et al., 2019) as significant predictors of suicidal ideation in African American adolescents. In the current study, both depression and hopelessness were significantly associated with ideation; participants with higher depression and hopelessness were more likely to be in the high ideation class. Depression was a consistent predictor of ideation class across all time points; however, hopelessness was only a significant predictor of ideation class at Time 2. This may be due to the increase in participants classified in the HI class at Time 2. At Time 2, approximately 13% of participants were classified in the HI class; this proportion was several points higher than the proportion of participants classified as HI at Time 1 (9.5%). Given the association between hopelessness and suicidal ideation in African American adolescents (Molock et al., 2006; Perkins & Hartless, 2002), such increases in suicidal ideation intensity suggests that hopelessness may be a critical target for preventive efforts. In this study, however, exposure to community violence was not associated with ideation. The research literature is not unequivocal regarding the association of CVE to suicide risk for African American adolescents and this study further confirms that more research in this area is needed. While some studies have found direct associations between CVE and ideation (e.g., Farrell et al., 2019), others have found that CVE indirectly affects ideation through other individual-level factors, like depression and aggression (Bennett & Joe, 2015; Lambert et al., 2008). It may be that contextual factors, such as CVE, have their effects through other individual-level factors.

The findings of the present study support that suicidal ideation in African American adolescents is highly prevalent. The likelihood of continued high suicidal ideation across assessment points was often lower than the likelihood of transitioning to a low ideation state, suggesting that experiences of high ideation were not protracted for most participants. However, prevention efforts will need to identify youth who are experiencing high ideation with urgency, given the heightened risk status of these youth.

The findings of this study have important implications for suicide risk assessment and preventive intervention strategies for African American adolescents. The identification of suicidal ideation classes may help to target mental health interventions and resources to the subgroup of adolescents most in need of support. Our findings indicated that approximately 10% of adolescents would be classified in the HI group at any given time, but that a substantially larger subset of African American adolescents would experience high ideation at some point during ninth or tenth grade. Identification of students with high ideation may help to target limited school-based mental health resources to those most in need. In addition, we found that the probability of staying in the HI group across two adjacent time points ranged from 0.29 to 0.55. Rapid intervention for students with high ideation may work to decrease the probability of prolonged high levels of ideation and progression to more serious risk for self-harming behaviors. These findings suggest the need for increased universal screening for suicide risk and targeted support and intervention for those identified as at higher risk. Further, identification of transitions to higher levels of ideation may be an important indicator of suicide risk (Thompson et al., 2009). Transitions from low to high levels of ideation may indicate important changes in individual and/or contextual factors that convey greater risk (e.g., depression), some of which may be amenable to change through intervention. Identification of predictors of consistently high levels of ideation may further inform preventive intervention, as well as traditional secondary and tertiary intervention. Chronically high levels of ideation may prompt the use of more intensive and/or longer-term strategies.

Both assessment and intervention strategies will need to address the unique etiology of suicidality in African American adolescents (Goldston et al., 2008). This includes the identification of changes in depressive symptoms or hopelessness, which may be associated with higher levels of ideation and, particularly for hopelessness, may be indicators of a transition from passive to active suicidal ideation. Given the ubiquity of suicidal ideation in the current sample, efforts to enact culturally-relevant, universal prevention programming for African American adolescents may help to mitigate this problem. Universal preventive interventions particularly developed to reduce symptoms of depression and hopelessness in African American adolescents may have downstream effects of reducing suicidal ideation.

There were study strengths and limitations that are important to consider. This study utilized a community sample of African American adolescents with varying levels of suicidal ideation. This allowed for modeling patterns in suicidal ideation from a universal perspective, rather than modeling ideation among individuals that indicated somewhat high levels of disturbance or ideation. Additionally, the use of LTA allowed for a more granular view of suicidal ideation patterns and transitions. Study limitations include the sample size, which limited our ability to examine other potential covariates, including the potential moderating role of gender. We note that study findings may not be generalizable to all African American adolescents. As participants from this study are in-school youth who were drawn from one large Midwestern city, results may not be generalizable to out-of-school African American adolescents or those living in other environments. Future studies may benefit from including African American adolescents in rural, as well as urban, environments to determine whether there are geographic differences in patterns of ideation. Additionally, since data collection occurred during regular school hours, we were unable to assess students who had dropped out of school or infrequently attended school. Youth who are disengaged with the school system may have differing levels of risk than youth engaged with school. Recognizing that African American adolescents are not a monolithic group, future research that includes the above-mentioned youth may draw attention to new and unique needs for particular subgroups of African American adolescents.

In conclusion, our findings add to the growing concern regarding suicidality and the significant rise in suicide rates among African American adolescents (Price & Khubchandani, 2019). Suicidal ideation is linked to future suicide attempts in African American adolescents (Musci et al., 2016), and these attempts can be fatal (Price & Khubchandani, 2019). As suicidal ideation has been shown to be associated with a greater likelihood of completed suicide, to a degree similar to the association between suicidal behaviors and completed suicide (Large et al., 2021), ideation is a cause for considerable concern. Targeting suicidal ideation for preventive intervention in the ninth grade or earlier may help to prevent subsequent adverse sequelae in African American youth, as well as help African American youth experiencing ideation to cope with associated stressors and return to no ideation or a low-ideation state. Our results underscore the acute need for the development and dissemination of culturally relevant preventive intervention efforts that target suicidal ideation in African American adolescents.

## Figures and Tables

**TABLE 1:** Descriptive statistics for suicidal ideation indicators and covariates (*n* = 99).

	Wave 1	Wave 2	Wave 3	Wave 4
	*M* (*SD*)	*M* (*SD*)	*M* (*SD*)	*M* (*SD*)
**Indicators**				
“I had thoughts about death”	0.46 (0.80)	0.49 (0.89)	0.45 (0.71)	0.49 (0.75)
“I felt my family and friends would be better off if I were dead”	0.37 (0.83)	0.30 (0.71)	0.31 (0.70)	0.25 (0.60)
“I thought about killing myself”	0.21 (0.59)	0.24 (0.56)	0.20 (0.59)	0.25 (0.69)
“I felt that I would kill myself if I only knew a way”	0.18 (0.56)	0.26 (0.74)	0.18 (0.58)	0.22 (0.68)
**Covariates**				
Depression	16.96 (10.21)	17.68 (9.46)	16.70 (9.25)	15.98 (9.12)
Hopelessness	3.37 (3.18)	4.53 (3.54)	4.15 (3.45)	4.16 (3.32)
Community violence exposure	25.32 (14.36)	23.24 (19.13)	20.21 (17.91)	23.83 (18.72)

**TABLE 2:** Model fit indices for measurement models.

	1 Class	2 Classes	3 Classes	4 Classes
**T1**				
Log likelihood	−682.19	−504.19	−445.262	−318.88
BIC	1404.981	1074.35	981.88	754.48
VLMR-LRT	N/A	0.004	0.199	0.416
BLRT	N/A	0.000	0.000	0.000
**T2**				
Log likelihood	−688.43	−497.52	−403.01	−369.76
BIC	1416.62	1059.64	895.47	853.84
VLMR-LRT	N/A	0.067	0.301	0.157
BLRT	N/A	0.000	0.000	1.000
**T3**				
Log likelihood	−467.56	−299.74	−219.31	−183.49
BIC	973.35	661.60	524.64	476.90
VLMR-LRT	N/A	0.135	0.784	0.740
BLRT	N/A	0.000	0.000	0.000
**T4**				
Log likelihood	−418.78	−265.04	−232.77	−188.16
BIC	874.64	590.32	548.97	482.92
VLMR-LRT	N/A	0.201	0.484	0.716
BLRT	N/A	0.000	0.000	0.000

*Note*: T1 = time point 1; T2 = time point 2; T3 = time point 3; T4 = time point 4. For Waves 2–4, in the 3- and 4-class solutions, the best loglikelihood was not replicated, suggesting that standard errors may not be trustworthy. Additionally, for waves 3 and 4, the 3- and 4-class models each had one class that represented less than 5% of the overall sample.

Abbreviations: BIC, Bayesian Information Criteria; BLRT, Bootstrapped Likelihood Ratio Test; VLMR-LRT, Vuong-Lo-Mendell-Rubin Likelihood Ratio Test.

**TABLE 3:** Percentages of participants in each suicidal ideation class across waves.

	T1	T2	T3	T4
LI	90.50%	86.68%	88.90%	87.89%
HI	9.50%	13.32%	11.10%	12.11%

*Note*: T1 = time point 1; T2 = time point 2; T3 = time point 3; T4 = time point 4.

Abbreviations: HI, high suicidal ideation; LI, low suicidal ideation.

**TABLE 4:** Transition probabilities for the 2-class LTA model with covariates.

	LI	HI
**T1**	**T2**	
LI	0.915	0.085
HI	0.452	0.548
**T2**	**T3**	
LI	0.920	0.080
HI	0.708	0.292
**T3**	**T4**	
LI	0.922	0.078
HI	0.523	0.477

*Note*: T1 = time point 1; T2 = time point 2; T3 = time point 3; T4 = time point 4. Table depicts transition probabilities for adjacent time points. Earlier time points are represented in rows, and later time points are represented in columns.

Abbreviations: HI, high suicidal ideation; LI, low suicidal ideation.

**TABLE 5:** Percentages of participants in possible transition patterns of 2-class LTA model.

T1	T2	T3	T4	Percentage (%)
LI	LI	LI	LI	72.73
LI	LI	LI	HI	4.04
LI	LI	HI	LI	4.04
LI	LI	HI	HI	1.01
LI	HI	LI	LI	4.04
LI	HI	LI	HI	2.02
LI	HI	HI	LI	0.00
LI	HI	HI	HI	2.02
HI	LI	LI	LI	4.04
HI	LI	LI	HI	0.00
HI	LI	HI	LI	0.00
HI	LI	HI	HI	1.01
HI	HI	LI	LI	1.01
HI	HI	LI	HI	1.01
HI	HI	HI	LI	2.02
HI	HI	HI	HI	1.01

*Note*: T1 = time point 1; T2 = time point 2; T3 = time point 3; T4 = time point 4.

Abbreviations: HI, high suicidal ideation; LI, low suicidal ideation; LTA, latent transition analysis.

**TABLE 6:** Odds ratios and *p* values for time-varying covariates in LTA 2-class model, with HI class as referent group.

Variable	Odds ratio	Standard error	*p* value
T1			
Depression	0.89	0.03	<.001
Hopelessness	0.98	0.14	.853
Community violence exposure	0.99	0.04	.681
T2			
Depression	0.90	0.03	.003
Hopelessness	0.72	0.08	<.001
Community violence exposure	1.00	0.02	.961
T3			
Depression	0.81	0.07	.006
Hopelessness	1.00	0.12	.977
Community violence exposure	0.94	0.03	.093
T4	0.91	0.04	.032
Depression			
Hopelessness	0.90	0.10	.320
Community violence exposure	0.97	0.02	.094

*Note*: T1 = time point 1; T2 = time point 2; T3 = time point 3; T4 = time point 4.

Abbreviations: HI, high ideation; LTA, latent transition analysis.

## Data Availability

Data included in this study may be made available upon reasonable request to the corresponding author.

## Highlights

- We examined longitudinal patterns of suicidal ideation in African American high school students
- We identified two classes of suicidal ideation: Low ideation and High ideation
- About 30% of participants experienced high ideation at some point during ninth or tenth grade
- Depression and hopelessness were associated with an increased risk for high suicidal ideation
- Understanding patterns of suicidal ideation may inform effective preventive interventions
